# Supplementary material for: Induced alpha and beta electroencephalographic rhythms covary with single-trial speech intelligibility in competition
Source: Sci Rep. 2023 Jun 23;13:10216. doi: 10.1038/s41598-023-37173-2 (PMC10290148; doi:10.1038/s41598-023-37173-2)
Supplement: Supplementary file 5 — Supplementary Information 5. [file 41598_2023_37173_MOESM5_ESM.docx]

**Induced Alpha And Beta Electroencephalographic Rhythms Covary With Single-Trial Speech Intelligibility In Competition**

Vibha Viswanathan [1,*], Hari M. Bharadwaj [2], Michael G. Heinz [3], Barbara G. Shinn-Cunningham [1]

[1] Neuroscience Institute, Carnegie Mellon University, Pitttsburgh, PA 15213. [2] Department of Communication Science and Disorders, University of Pittsburgh, Pitttsburgh, PA 15260. [3] Department of Speech, Language, and Hearing Sciences, Purdue University, West Lafayette, IN 47907.

*Correspondence: vibhavis@andrew.cmu.edu

**Supplementary Information**

| 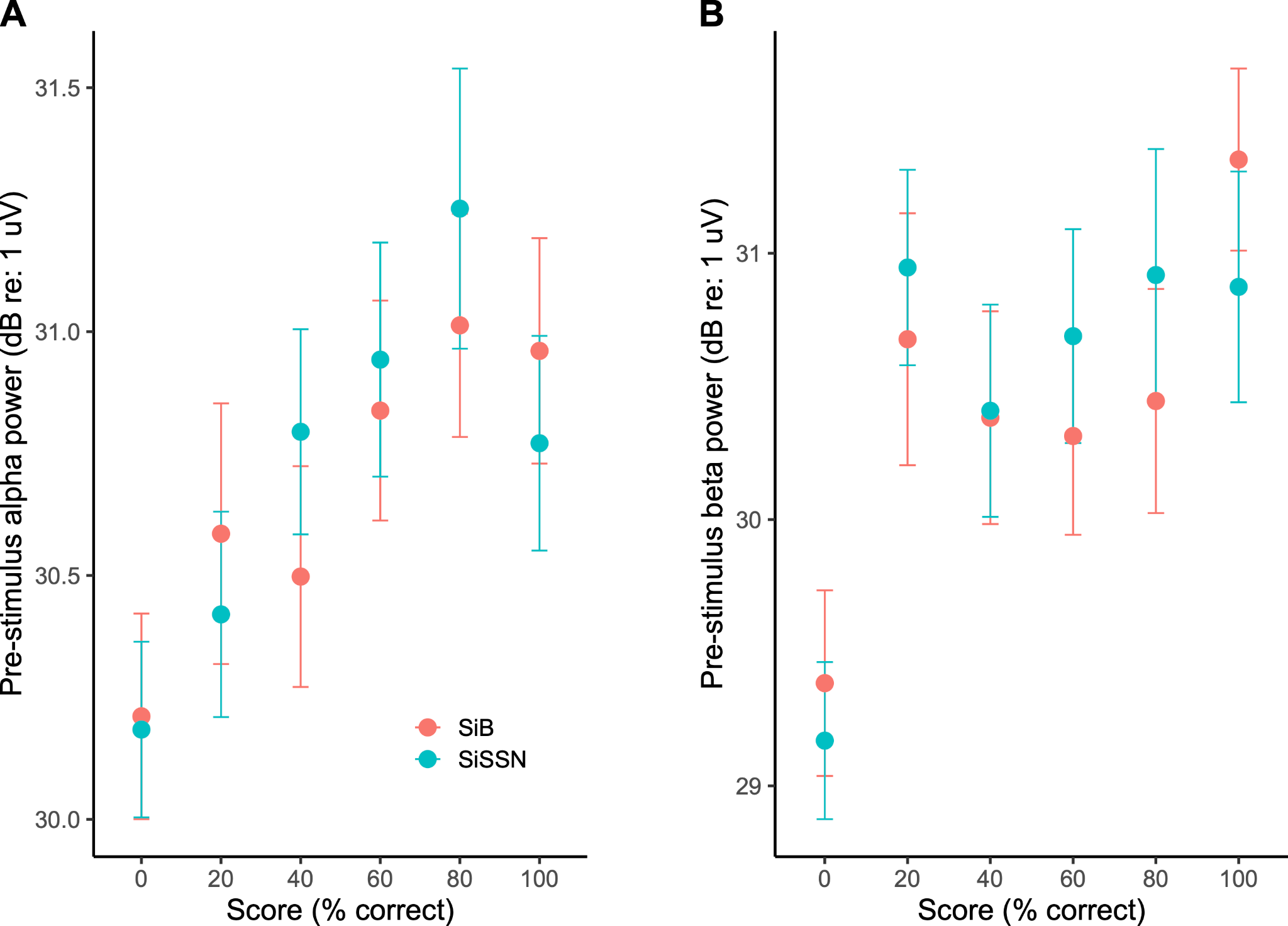 |
| --- |
| **Supplementary Figure S1.** Pre-stimulus alpha (A) and pre-stimulus beta (B) power [mean and standard error of the mean (STE)] versus percent-correct score in different trials across subjects. Data are shown separately for each experimental condition (SiB versus SiSSN). |

| 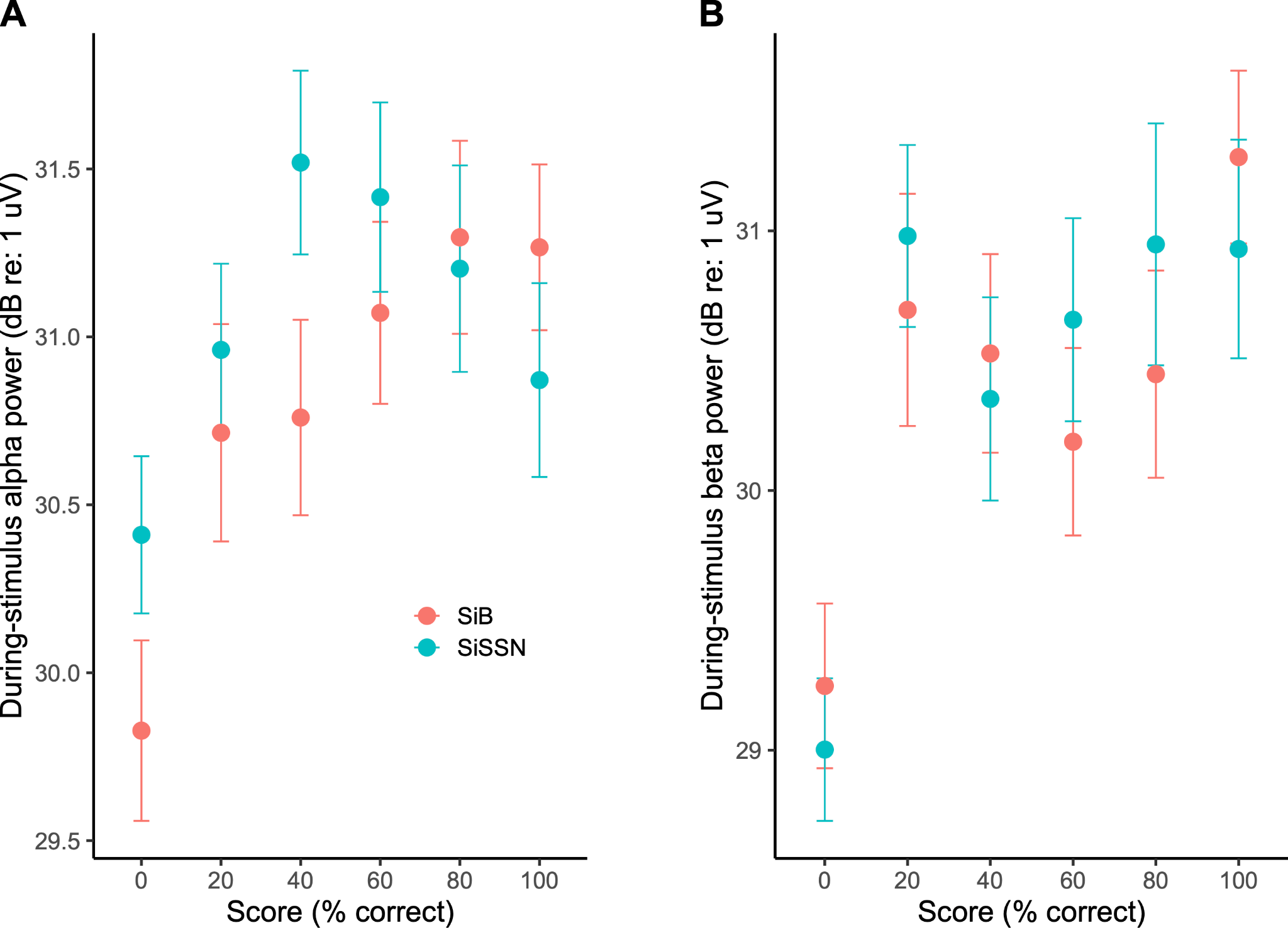 |
| --- |
| **Supplementary Figure S2.** During-stimulus alpha (A) and during-stimulus beta (B) power (mean and STE) versus percent-correct score in different trials across subjects. Data are shown separately for each experimental condition (SiB versus SiSSN). |

| **Supplementary Table S1.** Analysis of deviance table (Type II tests) for the multinomial linear regression analysis to test whether during-stimulus alpha power contributes additionally to predicting percent-correct score over the contribution of pre-stimulus alpha power alone, and vice-versa. | | | |
| --- | --- | --- | --- |
| R code (uses the “nnet” package):  model <- multinom(formula = percentcorrect ~ duringstimulus_alpha + prestimulus_alpha + condition, data = data)  Anova(model) | | | |
|  | Chi-square | Degree of freedom | Probability(>Chi-square) |
| duringstimulus_alpha | 13.912 | 5 | 0.01618 |
| prestimulus_alpha | 9.933 | 5 | 0.07717 |
| condition | 38.673 | 5 | 2.763e-07 |

| **Supplementary Table S2.** Analysis of deviance table (Type II tests) for the multinomial linear regression analysis to test whether during-stimulus beta power contributes additionally to predicting percent-correct score over the contribution of pre-stimulus beta power alone, and vice-versa. | | | |
| --- | --- | --- | --- |
| R code (uses the “nnet” package):  model <- multinom(formula = percentcorrect ~ duringstimulus_beta + prestimulus_beta + condition, data = data)  Anova(model) | | | |
|  | Chi-square | Degree of freedom | Probability(>Chi-square) |
| duringstimulus_beta | 12.931 | 5 | 0.02404 |
| prestimulus_beta | 4.089 | 5 | 0.53674 |
| condition | 38.371 | 5 | 3.178e-07 |
